# In-situ growth of MnO_2_ crystals under nanopore-constraint in carbon nanofibers and their electrochemical performance

**DOI:** 10.1038/srep37368

**Published:** 2016-11-21

**Authors:** TrungHieu Le, Ying Yang, Liu Yu, Zheng-hong Huang, Feiyu Kang

**Affiliations:** 1 State Key Laboratory of Control and Simulation of Power System and Generation Equipments, Tsinghua University, Beijing 100084, China; 2 Laboratory of Advanced Materials, Department of Materials Science and Engineering, Tsinghua University, Beijing 100084, China

## Abstract

Growing MnO_2_ nanocrystals in the bulk of porous carbon nanofibers is conducted in a KMnO_4_ aqueous solution aimed to enhance the electrochemical performance of MnO_2_. The rate of redox reaction between KMnO_4_ and carbon was controlled by the concentration of KMnO_4_ in a neutral solution. The MnO_2_ nanoparticles grow along with (211) crystal faces when the redox reaction happens on the surface of fibers under 1D constraint, while the nanoparticles grow along with (200) crystal faces when the redox reaction happens in the bulk of fibers under 3D constraint. The composite, where MnO_2_ nanoparticles are formed in the bulk under a constraint, yields an electrode material for supercapacitors showing good electron transport, rapid ion penetration, fast and reversible Faradaic reaction, and excellent rate performance. The capacitance of the composite electrode could be 1282 F g^−1^ under a current density of 0.2 A g^−1^ in 1 M Na_2_SO_4_ electrolyte. A symmetric supercapacitor delivers energy density of 36 Wh kg^−1^ with power density of 39 W kg^−1^, and can maintain 7.5 Wh kg^−1^ at 10.3 kW kg^−1^. It exhibits an excellent electrochemical cycling stability with 101% initial capacitance and 95% columbic efficiency even after 1000 cycles of charge/discharge.

Depending on the specific charge storage mechanisms, capacitances are divided into double layer capacitance (EDLC), relying on the high surface area of electrode materials to adsorb charges at the electrode/electrolyte interfaces, and pseudo-capacitance, relying on Faradaic reversible redox reactions near the surface of the electrode^1^,^2^. The energy density is low for EDLCs as the ions physically accumulated on the interfaces are limited^3^. Conversely, the potential charge storage capability as pseudocapacitance is significantly higher than that of EDLCs, while its charge-discharge rate is often limited by the reaction kinetics. To improve the performance of the supercapacitor, recent researches are focusing on developing nanostructured composites of a carbon having high surface area and high conductivity with a transition metal oxide for electrode materials. Transition metal oxides (e.g. RuO_2_, MnO_2_, TiO_2_, Fe_2_O_3_, VO_x_, etc.) and conducting polymers (e.g. polypyrrole, polyaniline) are commonly coated or mixed with carbon materials to develop the composites^4^, by expecting both high specific energy and high power densities of the devices. Among the various transition metal oxides, MnO_2_ is suitable for supercapacitor applications because of its high theoretical specific capacitance, long cycle life, good durability, low cost, as well as environmental friendliness^5^,^6^. To make the composite of MnO_2_ with carbon scaffold is advantageous for improvement of poor electrical conductivity of MnO_2_. Therefore various carbon materials have been employed, activated carbons^7^,^8^,^9^, carbon nanotubes (CNTs)^10^,^11^,^12^, carbon nanofibers (CNFs)^13^,^14^,^15^,^16^,^17^,^18^,^19^, graphenes^20^,^21^,^22^,^23^ and graphene oxides^24^,^25^.

Homogeneous blending of MnO_2_ nanoparticles with carbon materials is still a challenging task. Since reversible redox reaction of MnO_2_ occurs only near the surface of the electrode because of small diffusion length of the electrolyte across the MnO_2_ as less than 10 nm, it has been greatly desirable to prepare nanostructured amorphous MnO_2_ to rise the utilization of its electrochemical activity. There are 4 representative methodologies for MnO_2_ nanocomposite preparation, including electrodeposition, electrostatic interaction assembly, *in-situ* redox deposition and chemical co-precipitation^26^. Based on these methods, several groups developed highly performance MnO_2_ nanocomposite electrode. Lang^27^ *et al*. showed that a supercapacitors made of nanoporous gold and nanocrystalline MnO_2_ resulted in a specific capacitance of 1145 F g^−1^ at 50 mV s^−1^. Fei group^28^ reported the reverse micro emulsion method to prepare 3–20 nm MnO_2_/3D RGO composites. The 3D RGO could provide channels for rapid ionic and electronic transport. The maximum specific capacitance of 709.8 F g^−1^ at 0.2 A g^−1^ of the MnO_2_ (66.4 wt%)/RGO composite. After 1000 cycles, 97.6% of the initial capacitance value can be maintained. Chen^29^ *et al*. prepared nanostructured MnO_2_/CNTs-sponge composite electrodes, which could give a high specific capacitance as 1230 F g^−1^ at 1 mV s^−1^. Based on the comparison in Table S1, the cost of the methods above is relatively high and the flexibility of the electrode is limited in most of the cases.

To develop a flexible and cost-effective method, Wang^30^ *et al*. and Ma^31^ *et al*. prepared MnO_2_/C composite based on an *in-situ* reaction between MnO_4_^−^ and carbon as the following reaction,


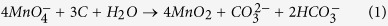


However, their attention was paid mainly on the influences of the length of reaction time^17^,^30^ and pH value of the solution^31^,^32^. The size of MnO_2_ obtained were submicrometers to micrometers. To improve the performance of MnO_2_/carbon fibers, Gao group^18^ and Qu group^19^ designed *in-situ* growth of MnO_2_ on the surface of activated carbon fibers. Gao^18^ reported that the composite displayed a capacitance of 117 F g^−1^. 116 F g^−1^ was kept after 3000 cycles. Qu^19^ *et al*. reported that the MnO_2_/PCNFs exhibited a specific capacitance of 520 F g^−1^ at 0.5 A g^−1^ and 92.3% retention of the initial capacitance after 4000 cycles in a 6 M KOH aqueous solution. It should be indicated that although porous carbon materials were used, both of them pointed out the growth of MnO_2_ on the surface of the fiber.

To further improve the capacitance of the MnO_2_/CNFs electrode, nano-MnO_2_ in the bulk of porous carbon nanofibers are needed. Amorphous carbon materials have strong reactivity with KMnO_4_. As the electrospun carbon fibers are amorphous^33^,^34^,^35^,^36^, there should be a competition of KMnO_4_ diffusion into the bulk and reaction of KMnO_4_ with carbon on the surface of the carbon fibers. If the diffusion time is shorter than the reaction time, the KMnO_4_ can diffuse into the bulk. Otherwise, the MnO_2_ crystals cover the surface of the fibers and block the diffusion route of KMnO_4_ into the bulk. Developing MnO_2_ with much smaller sizes and high dispersion in carbon scaffold is urgently needed.

Herein, we have developed a controllable method for preparing high performance supercapacitor electrode with the nanocrystalline-MnO_2_ in the bulk of porous CNFs by controlling the kinetics of an *in-situ* redox reaction of KMnO_4_ under nanopore-constraint of CNFs (Eq. 1). The scaffold of porous CNFs, which was prepared from a mixture of polyimide (PI) and PVP via electrospinning^35^,^36^, acts as a matrix for electric double layer formation and a good electronic conductor to enhance the pseudocapacitive behavior of the nanocrystalline MnO_2_. More importantly, the nanopores provide volume for nanocrystalline MnO_2_ to grow. The pore volume limits the finial size of MnO_2_. Such kind of structure provides the enhanced utilization of MnO_2_ particles by easy diffusion of the electrolyte to the interfaces. In this work, reactants concentration and reaction temperature were considered to control the rate of chemical reaction mentioned above. A wide range of the concentration of KMnO_4_ was employed, *i.e*., 0.76 × 10^−4^, 1.52 × 10^−4^ and 7.60 × 10^−4^ M for the deposition of MnO_2_ nanoparticles onto the pristine CNFs. Since the relative concentration of KMnO_4_ in the solution can be expressed by 1:2:10, the MnO_2_/CNFs composites synthesized in these solutions are denoted as MC1, MC2 and MC10, respectively. For the comparison, the pristine CNFs without MnO_2_ loading was also used by denoting as MC0. Their structures, particularly those of deposited MnO_2_ on CNFs, and capacitive performance in Na_2_SO_4_ were characterized.

## Experimental results

### Structure

**Figure 1:**
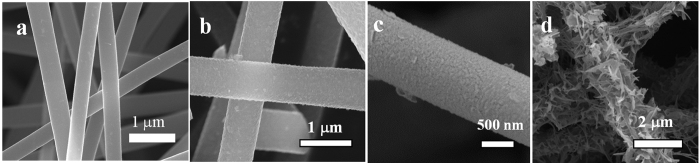
SEM images of the external surface of MnO_2_/CNFs composites (**a**) MC0, (**b**) MC1, (**c**) MC2 and (**d**) MC10.

Figure 1 shows the SEM images of the MnO_2_/CNFs composites. It is seen that the external surface of the pristine CNFs (MC0) is smooth and the diameters of fibers are in the range of 300–600 nm (Fig. 1a). After loading MnO_2_ by a redox reaction with KMnO_4_, the appearance of CNFs is in sharp contrast to that of MC0, particularly that of MC10. For those CNFs reacted with KMnO_4_ in a low concentration, MC1 and MC2, the surfaces of CNFs became rough and nanoparticles of MnO_2_ can be recognized, as shown in Fig. 1b and c, respectively. For MC10, which is prepared in a high concentration of KMnO_4_, large-sized needle-like crystals of MnO_2_ are formed on the fiber surface (Fig. 1d).

**Figure 2:**
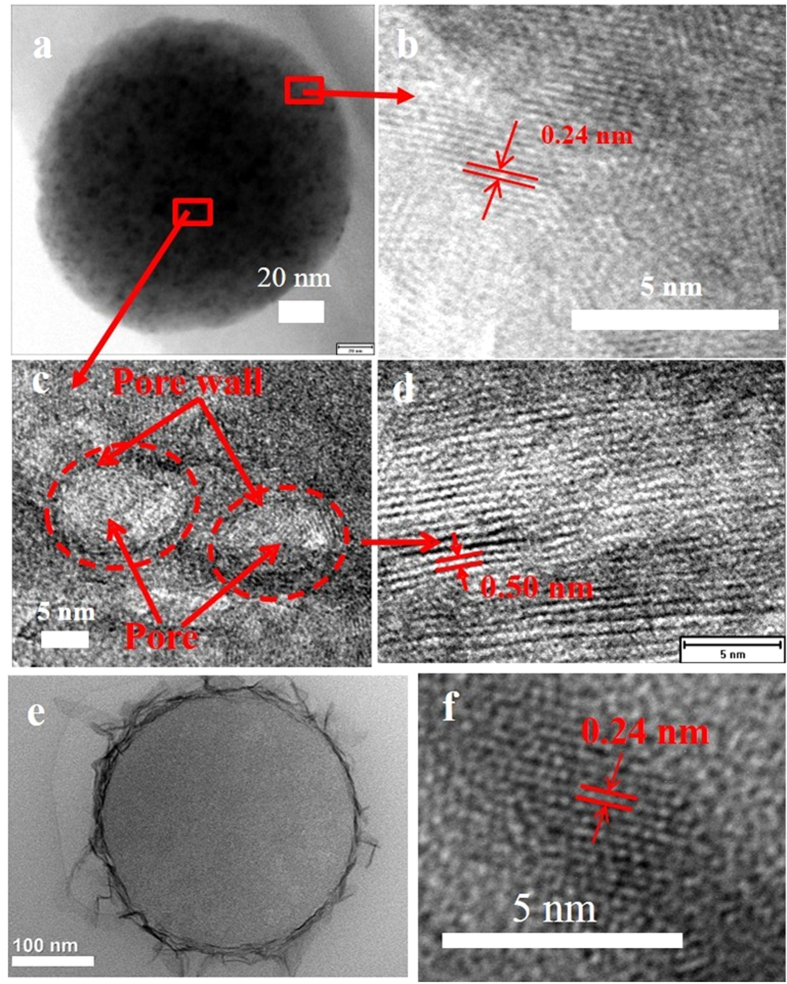
(**a**) TEM image of the cross-section of MC2, HRTEM images of the (**b**) external part of MC2 cross-section, (**c,d**) inner part of MC2 cross-section, (**e**) cross-section of MC10, (**f**) external part of MC10 cross-section.

Detailed transmission electron microscope (TEM) observations were carried out by using thin slices of the fibers. Figure 2a shows TEM image of the cross-section of a MC2 fiber. High resolution TEM (HRTEM) images of an external part and an inner part of the cross-section are shown in Fig. 2b and c, respectively. In these HRTEM images of MC2, lattice fringes are clearly seen, spacing of fringes being ca. 0.24 nm in the external part and ca. 0.50 nm in the inner part (Fig. 2d). In the HRTEM images of MC10 (Fig. 2e,f), lattice fringes are clearly seen, spacing of fringes being ca. 0.24 nm in the external part and no clear MnO_2_ were formed in the inner part.

**Figure 3:**
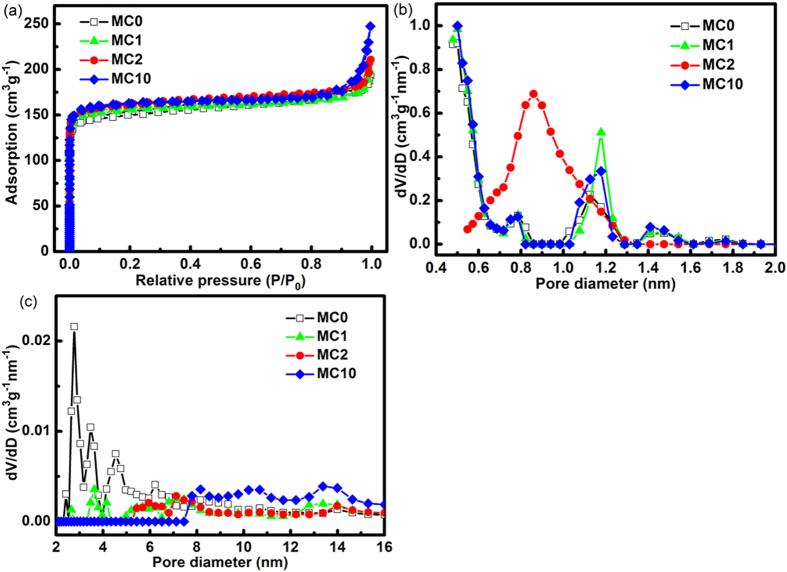
(**a**) N_2_ adsorption/desorption isotherms and (**b,c**) pore size distributions of MC samples.

N_2_ adsorption/desorption measurements were performed at 77 K to characterize the surface areas, pore volumes and the pore-size distributions of MC1, MC2, MC10 as well as pristine CNFs. All isotherms shown in Fig. 3a are typical IUPAC type I, suggesting the microporous structure^37^. As can be seen in Fig. 3b, MC10 and MC0 share a similar micropore distribution with peaks positioned below 0.4 nm and at around 1.2 nm in the pore size range of 0.5–2 nm, while a broad peak appeared in the range of 0.8–1.1 nm on MC2. As shown in Fig. 3c, the mesopores are detected in the range of 2–8 nm for MC0, but MC2 composites with MnO_2_ have only negligibly small amount of mesopores. When the concentration of KMnO_4_ increased to 0.12 g L^−1^ (MC10), pore-size distribution in micropore range becomes very similar to the pristine fibers (MC0), but the mesopores at 2–8 nm in MC0 disappear completely. On MC2 composite prepared by using the concentration of KMnO_4_ of 0.024 g L^−1^, very different distribution of micropore-size is observed.

In Table 1, surface areas and pore volumes are shown by dividing them into three regions, <0.7 nm, 0.7–2 nm and > 2 nm, together with BET surface area S_BET_ and total pore volume V_total_. With increasing KMnO_4_ concentration for the MnO_2_ loading onto MC0 fibers, both S_BET_ and V_total_ increase slightly. For MC2 composite, however, surface area and pore volume contributed by the pores with the sizes less than 0.7 nm, S_<0.7 nm_ and V_<0.7 nm_, decrease markedly and those by 0.7–2 nm increase markedly, although only slight increases in these parameters are detected for MC10.

**Figure 4:**
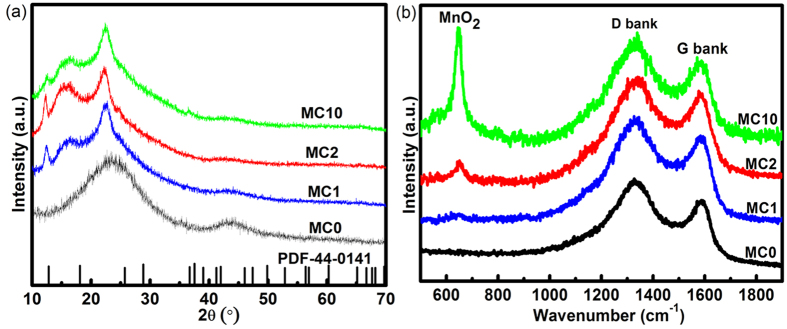
(**a**) XRD and (**b**) Raman spectra of MC samples.

X-ray diffraction patterns are shown for the composites, including MC0, in Fig. 4a. For MC0, there are two broad peaks at 2θ of around 26 and 44°, which can be ascribed to 002 and 10 diffraction of amorphous carbon^36^. For the composites loaded by MnO_2_ (MC1, MC2 and MC10), the diffraction peaks of MnO_2_ at around 12, 17, 24, 37 and 66° in 2θ, of which index are 110, 200, 220, 211 and 002, respectively (JCPDS No. 44–0141).

Raman spectra of the composites are shown in Fig. 4b. The D- and G-bands of amorphous carbon^38^ are observed for the composites as well as the pristine MC0, of which intensity ratio I_D_/I_G_ was calculated to be 2.7, indicating the turbostratic structure of the CNFs. For the composites, one additional band from MnO_2_ is observed at 646 cm^−1,^ which can be assigned to the symmetric stretching vibration of Mn-O in the MnO_6_ groups^25^,^39^. It should be noted that the bands belonging to MnO_2_ become more and more distinct with the increase of KMnO_4_ concentration.

**Figure 5:**
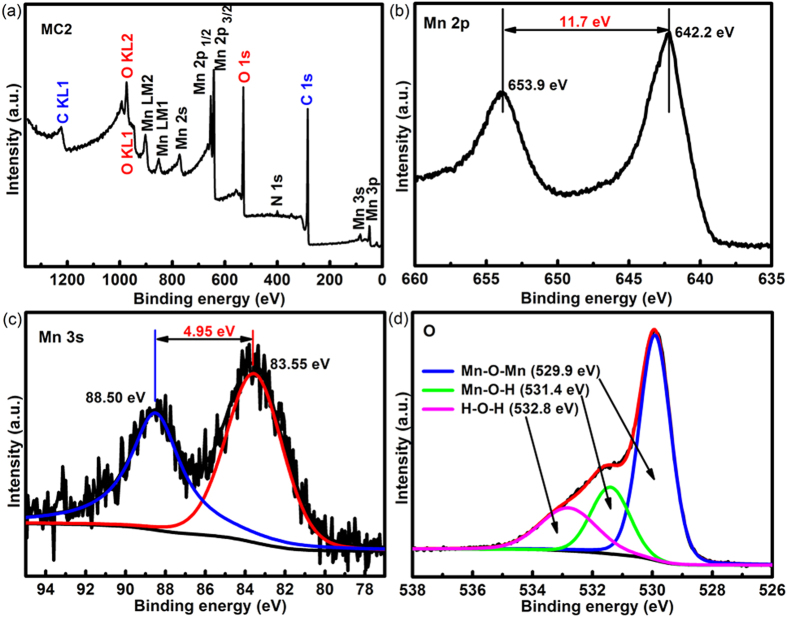
XPS spectra of MC2 composites (**a**) elements composition, binding energy separation of (**b**) Mn 2p and (**c**) Mn 3 s, (**d**) the intensities of Mn-O-Mn and Mn-OH.

To investigate the chemical composition and chemical states of various elements in the composites, XPS analysis was performed. The spectrum in Fig. 5a reveals the existence of O, Mn and C in the MC2 composite. From the binding energy separation of 11.7 eV between the peaks at 653.9 and 642.2 eV attributed to Mn2p1/2 and Mn2p3/2, respectively^40^ (Fig. 5b) and the separation energy of 4.95 eV of the Mn 3 s spin orbit doublet (Fig. 5c), an intermediate oxidation state of manganese was estimated^41^ to be around 3.7 for MC2. The valence of Mn was also estimated to be 3.66 from the intensities of the Mn-O-Mn (529.9 eV) and Mn-OH (531.4 eV) (Fig. 5d) according to the literature^42^,^43^. The intermediate valence state at around 3.7 in the composite MC2 seems to be benefiting the energy storage as a pseudocapacitance via the redox switching^41^.

### Electrochemical properties

**Figure 6:**
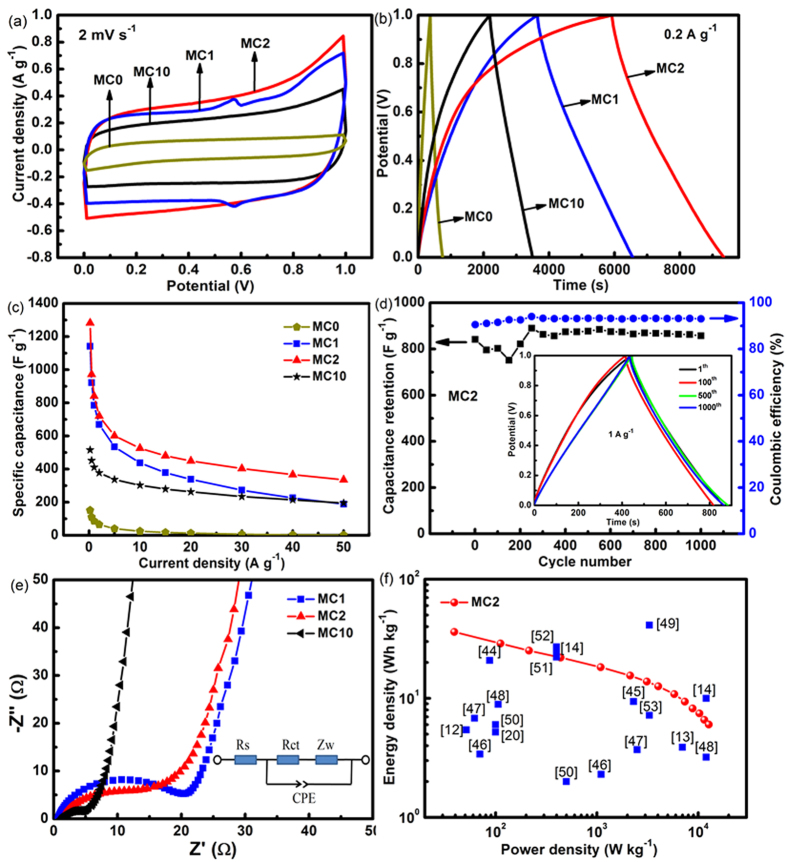
Electrochemical performances of MC composites (**a**) Cyclic Voltammetry (CV) curves of the MC composites at the scan rate of 2 mV s^-1^, (**b**) charge/discharge curves of the MC composites at the current density of 0.2 A g^-1^, dependences of (**d**) specific capacitance on current density for the MC composites and (**e**) specific capacitance and coulombic efficiency on cycle number for MC2 with the inset showing the charge/discharge curves in different cycles, (**f**) Nyquist plots of the EIS for MC1, MC2 and MC10 composites with the inset showing the equivalent circuit used, (**g**) Ragone plots for the present MC2 composites in comparison with published data.

Cyclic voltammetry (CV) curves in 1 M Na^2^SO_4_ electrolyte with different scan rates from 2 to 100 mV s^−1^ are shown for the composites in Fig. 6a and Supplementary Fig. S1. The composite MC2 is expected to have the highest capacitance.

Galvanostatic charge/discharge curves measured with the current density of 0.2 and 10 A g^−1^ are summarized for the composites in Fig. 6b and Supplementary Fig. S1, respectively. For the composites MC2 and MC10, the curves are not straight during both charging and discharging, revealing certain contribution of pseudocapacitance due to MnO_2_. The specific capacitance calculated from these curves with 0.2 A g^−1^ was 1282 F g^−1^ for MC2, while 151 and 515 F g^−1^ for MC0 and MC10, respectively. When increasing the charging/discharging current density to 10 A g−1, all curves present representative relatively linear voltage-time relationship with quasi-symmetric triangular shapes compare to the charging/discharging curve at 2 A g−1, revealing a remarkable reversibility, which indicate the capacity is mainly contributed by physically adsorption under larger current density.

The dependences of specific capacitance on current density are plotted for the composites in Fig. 6c. It is seen that the specific capacitance decreases with the increase of current density, abruptly up to 10 A g^−1^ and then gradually. For MC2, the specific capacitance remains as high as 335 F g^−1^ at a current density of 50 A g^−1^, the capacity retention ratio being 26%. The capacitance retention with the current density of 1 A g^−1^ is shown for MC2 in Fig. 6d, the capacitance fluctuating during the first 200 cycles and then being stabilized at about 100%. The capacitance fluctuation in the beginning is probably due to the low utilization of inner MnO_2_ as the limitation of electrolyte diffusion into the bulk of the fibers. After 200 cycles, as the electrodes gradually soaked by electrolyte, more MnO_2_ are involved in the charge storage process. The retention ratio start to increase, then slowly exceed its initial value. Electrodes display an ideal cycling stability with full permeation of electrolyte after approximate 500 cycles, reaching a remarkable capacitance retention of 101%. 95% Coulombic efficiency remained after 1000 continuous cycles measured using the galvanostatic charge-discharge technique.

The electrochemical impedance spectroscopy (EIS) of the composite is displayed in Fig. 6e, the equivalent circuit diagram being in the inset. The equivalent circuit includes bulk solution resistance Rs, charge transfer resistance Rct, pseudo-capacitance Cp due to the redox reactions of MnO_2_ and Warburg impedance Zw. It is seen that all composite electrodes exhibit the nearly vertical line along the imaginary axis in the low-frequency region, revealing an ideally capacitive behavior of the electrode materials due to the fast and reversible Faradic reaction of nano-MnO_2_. It should be noted that the slope is larger with MC10 than MC2. The Rs for composite electrodes remained almost the same because this parameter is insensitive to the electrode surface. In the high frequency region, the charge transfer resistance (Rct) was 11.5 and 2.5 Ω for MC2 and MC10, respectively, which seems to depend strongly on the concentration of KMnO4 during loading of MnO_2_.

Ragone plot is presented to characterize the MnO_2_/CNFs composites in Fig. 6f together with the data published. It is noteworthy to mention that energy density of MC2 is 36 Wh kg^−1^ with power density of 39 W kg^−1^, and can maintain 7.5 Wh kg^−1^ at 10.3 kW kg^−1^. Comparing the present composite MC2 with those published on electrospun carbon fibers and activated carbons, the performance of MC2 surpasses most of MnO_2_/carbon composites reported^12^,^13^,^14^,^20^,^44^,^45^,^46^,^47^,^48^,^49^,^50^,^51^,^52^,^53^. With regard to active materials, the energy density can be calculated through the following equations:


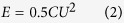


Two strategies are commonly used to develop electrode with high energy density under high power density. (1) One is to extend the potential window in aqueous electrolytes via designing an asymmetric configuration^50^,^54^,^55^ or using special electrolyte with high potential window^14^,^49^,^56^,^57^. By using Na_2_SO_4_ aqueous electrolyte, the potential window of the supercapacitor can be 1.6–2.2 V^14^,^49^,^57^. The achieved performance curve is overlapped with our results, although the MnO_2_/C composite performance is about 292 F g^−1^ (2.5 A g^−1^). (2) The other is designing electrode with high specific capacitance. Many efforts have been done to design the host material micro-architecture for loading nanoscopic MnO_2_ to balance electron and ion transport inside the composites, as well as mass loading. Except the energy and power density shown in Fig. 6(f), more details for comparison are shown in Table S3. Several groups reported that, shorter redox reaction time of KMnO_4_ and carbon in neutral solution can help to achieve thinner MnO_2_ with higher specific capacitance and high energy density^12^,^13^,^30^. Larger mass loading accompanies with lower specific capacitance in most of the cases. With porous structure, more nano-sized MnO_2_ can be loaded^50^. High utilization of active materials can be achieved by combining the homogeneous deposition of MnO_2_ nanocrystals in the bulk because the rapid intercalation/deintercalation of Na^+^ on the surface layer of MnO_2_ has been widely accepted as the charge-storage mechanism in mild electrolytes. The combination of MnO_2_ and porous CNFs can achieve excellent capacitive performance. More interface between the MnO_2_/conductive material can improve the MnO_2_ utilization mass ratio. In most of the cases shown in Fig. 6d, The MnO_2_ crystal size is tens of nanometers. Although the MnO_2_ is exposed to the electrolyte, the interface between the MnO_2_ and carbon is limited. The interface between 3D contrained nano-MnO_2_ and porous CNFs has been enhanced dramatically while the resistance of the matrix increases with more loading of nano-MnO_2_ in the bulk of CNFs. So it is a trade-off between the energy density and power density. Furthermore, comparing CNFs with activated carbons and porous carbon spheres, the pores in CNFs are shallow ones since the fiber diameter is only sub-micros while the pores in activated carbons or carbon spheres are deep and narrow. The electrolyte penetration resistance of CNFs should be smaller than that of activated carbons. So, the electron transport loops and the diffusion loops of cations in most of the cases are longer than the structure shown in this work, which is related to the power performance of an electrode.

## Discussion

At the interface of electrode and Na_2_SO_4_ electrolyte, two charge storage mechanisms of MnO_2_ are proposed, including surface adsorption/desorption and bulk insertion/extraction of Na^+^, which are showed below^42^:


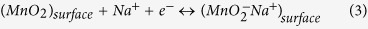



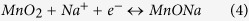


To get more insights into the advantages of the MC2 electrode, a high resolution TEM has been used. Figure 2(c–e) clearly revealed that MnO^2^ crystals with an interlayer spacing of about 0.24 nm were composed of on the external surface of the fibers, corresponding to α-MnO_2_ (211) crystal faces, while MnO_2_ with an interlayer spacing of about 0.5 nm were deposited on the inner surface of the pores at the central part of fibers, exhibiting the character of α-MnO_2_ (200) crystal faces. The results are in good agreement with XRD information. The results indicate that MnO_2_ nanoparticles grow along with (211) crystal face when the redox reaction between KMnO_4_ and carbon happens on the surface of CNFs, in other words, under an un-limited 1D constraint; while MnO_2_ nanoparticles grow along with (200) crystal face when the redox reaction happens inside of mesopores, under a strong 3D constraint from the inner surface of pores.

**Figure 7:**
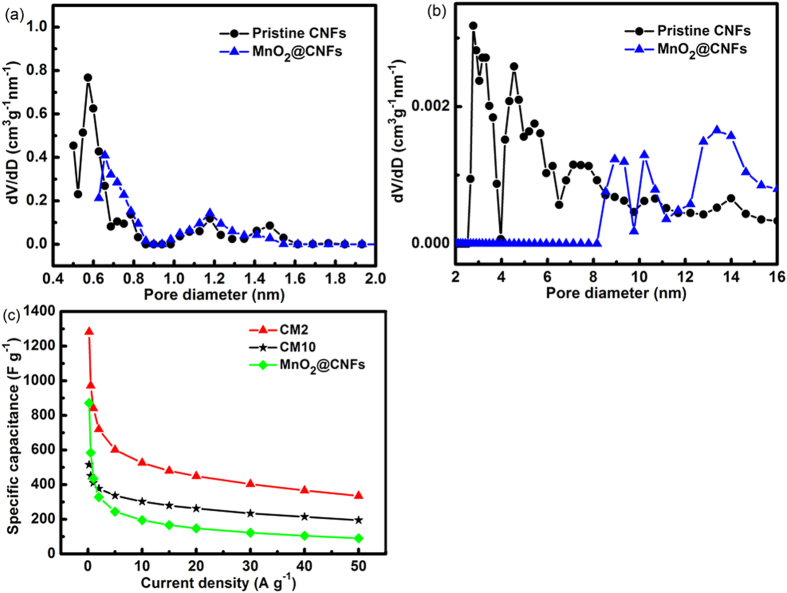
(**a,b**) Pore-size distributions of the pristine CNFs and ones loaded with MnO_2_ under the same conditions as MC2 composites, (**c**) dependences of specific capacitance on current density for MnO_2_/PVP induced porous CNFs and MnO_2_/PVP free CNFs electrodes.

To figure out the affection of porous structure in CNFs on in-situ reaction between KMnO_4_ and carbon to form MnO_2_, the electrospun CNFs prepared from PI without PVP were used as scaffold for MnO_2_ by the same condition as MC2 (KMnO_4_ concentration of 0.024 g L^−1^). Pore-size distributions for the pristine (MnO_2_-unloaded) and MnO_2_-loaded CNFs are shown in Fig. 7(a,b), displaying an increase of pores having the size of 1.0–1.5 nm by sacrificing the mesopores having 2–8 nm sizes. The change in pore structure in the CNFs with MnO_2_-loading is very similar to that happened in the composite MC2 (Fig. 3). Comparing to the pristine CNFs, the pore volume contributed from pores smaller than 0.7 nm disappear, a big increase of pore volume from pores in the range of 0.7–2 nm appeared and a decrease of pore volume happens with the pore volume contributed from pores larger than 2 nm for MC2 (Fig. 3) and also another CNFs (MnO2-loaded in Fig. 7). This should be related to the PVP induced pores. Porous CNFs has a larger pore volume and more defects. The defects in the bulk can prove more active-edges for the reaction with KMnO_4_ to produce MnO_2_ in the 3D constrains.

Based on the pore distribution and TEM images, we could conclude that KMnO_4_ penetrated to the pores situated inside of the porous CNFs. The MnO_2_ nanoparticles formed inside of the pores covered the inner surface of the pores. So, the pores smaller than 0.7 are blocked by MnO_2_ either deposited at the entrances of pores or formed in the pores, resulting in the disappearance of the peaks for the pores smaller than 0.7 nm. The increase of pore volume in the range of 0.7–1.2 nm should be related to the MnO_2_ nanoparticles formed in the pores larger than 2 nm.

Based on the CV test and galvanostatic charge/discharge results, it could be seen that the performances of MnO_2_/PVP induced porous CNFs electrode are all better than MnO_2_/PVP free CNFs electrode. The PVP free samples MnO_2_@CNFs shows better capacity at smaller current density and the lower capacity at larger current density than MC10 (Fig. 7c). The results indicate that MnO_2_ with low crystalline was produced in low concentration KMnO_4_, which bring us a higher utilization rate of MnO_2_ in the supercapacitor performances.

As shown in Fig. 6a, Supplementary Fig. S1 and Fig. 6e of the CV test and EIS results, MC2 shows larger capacitance than MC10 while the resistance of MC10 is much lower than MC2 and MC1. This should be related to formation of MnO_2_ in the bulk of the electrode.

With a high concentration of KMnO_4_, the redox reaction is reasonably supposed to occur very fast with amorphous carbons, and so MnO_2_ nanoparticles are formed on the surface of the fibers, which seems to prevent the penetration of KMnO_4_ into the bulk of the fiber. The morphology of the electrode is quite different with that in a low concentration KMnO_4_, even in the form of flaks, as shown in Fig. 1d for MC10. The crystallinity of MnO_2_ produced in a high concentration KMnO_4_ is lower than that of MnO_2_ produced in low concentration KMnO_4_, which is proved by XRD as shown in Fig. 4a. In MC10, the pore volume does not change too much. Such a pore-size distribution suggested that MnO2 deposition occurs only on the surface of the CNFs. The resistance of such kind of structure should be low which is also approved by EIS data.

When the time for the diffusion of KMnO_4_ into pores is the same order of magnitude with the time for the redox reaction of KMnO_4_ with carbon, MnO_2_ nanocrystals can be precipitated in the pores of the fibers under a strong constraint. As MnO_2_ is an insulator, the bulk resistance should increase. Such kind of MnO_2_/C interface connection is very well. Therefore, the performance under larger current is also good.

## Conclusion

A 3D MnO_2_/C nano-composite with remarkable electrochemical behaviors can be on-site synthesized with porous carbon nanofibers as a scaffold. For MC2, the capacitance of the composite electrode could be 1282 F g^−1^ under a certain current density of 0.2 A g^−1^ in 1 M Na_2_SO_4_ electrolyte. It is noteworthy to mention that energy density of MC2 is 36 Wh kg^−1^ with power density of 39 W kg^−1^, and can maintain 7.5 Wh kg^−1^ at 10.3 kW kg^−1^. The key point of the work is to make the diffusion time of KMnO_4_ into the bulk of the porous carbon fibers and the redox reaction time of KMnO_4_ with carbon on the same order of magnitude by adjusting the concentration of KMnO_4_. The results indicate that the MnO_2_ growth along with (211) crystal face when KMnO_4_ and carbon redox reaction happens with an un-limited 1D constrain; while the MnO_2_ growth along with (200) crystal face when KMnO_4_ and carbon redox reaction happens with a comparable 3D constrain. The optimized KMnO_4_ concentration in our case should be 0.024 g L^−1^. The good capacitance performance of the MnO_2_/CNFs composite electrode is clearly attributed to its unique nanostructure. The porous CNFs serves as a highly conductive matrix for fast ion and electron transport, while the ultrathin MnO_2_ nanocrystals on the inner surface of the pores enable a short diffusion path of electrolyte and provide a more electrochemically active surface area for pseudocapacitance through fast and reversible Faradic reaction.

## Experimental section

### Materials

Pyromelliticdianhydride (PMDA) (Mw = 218.12 g mol^−1^, Sinopharm Chemical Reagent Co., Ltd), 4,4′-oxydianiline (ODA) (Mw = 200.24 g mol^−1^, Sinopharm Chemical Reagent Co., Ltd), polyvinyl pyrrolidones (PVP) (Mw = 1300,000 g mol^−1^, Sinopharm Chemical Reagent Co., LTD), N,N-dimethylformamide (DMF) (Xilong Chemical Co., Ltd) and potassium permanganate (KMnO_4_, 99.5%) (Sinopharm Chemical Reagent Co., Ltd) were used as received.

### Fabrication of carbon nanofibers

The electrospinning solution was prepared with PMDA, ODA and PVP in DMF with molar ratio of PMDA and ODA 1:1. The PVP was dissolved in the solution with mass ratio 20% (to total masses of PMDA and ODA). The blended solution was stirred at 0 °C for 24 h. Electrospinning parameters were set as follows: applied voltage of 20 kV, tip-to-collector distance of 20 cm and flow rate of 0.8 ml h^−1^. As-spun fibers were imidized and carbonized in a horizontal tubular furnace. Temperature of the furnace was first increased from room temperature to 300 °C at the rate of 3 °C min^−1^ and was maintained for 30 min in a flow of air for imidization. Then the temperature was increased to 900 °C at the same heating rate and was maintained for 1 h under N_2_ atmosphere for carbonization.

KMnO_4_ was dissolved into de-ionized water. The concentration of KMnO_4_ solution were prepared at 0.012 g L^−1^ (0.076*10^−3^ M), 0.024 g L^−1^ (0.152*10^−3^ M), and 0.12 g L^−1^ (0.76*10^−3 M).^ Composites derived from these solutions were labeled as MC1, MC2 and MC10, respectively.

The carbon fiber electrode was soaked into the KMnO_4_ solution and the weight ratio of CNFs to KMnO_4_ was set to 4:1. The CNFs was taken out until the purple color of KMnO_4_ solution had fade into golden brown. The mass loading remains to 10–11% for all samples. The MnO_2_/CNFs composites were rinsed by deionized water for several times, and finally dried at 80 °C for 10 h under vacuum condition.

### Characterization

The morphology of CNFs was investigated using a scanning electron microscope (SEM, LEO 1530). A high-resolution transmission electron microscopy (HRTEM) was employed to characterize the distribution and crystallization of the composites. The CNFs, pristine and MnO_2_-loaded, embedded in epoxy resin was cut into 20 nm slices using a microtome (EM UC6, Leica, Germany) with a diamond blade (DiATOME) and then placed onto a 300 mesh Cu grid for examination by TEM.

X-ray diffraction (XRD) pattern of the composites was examined using D/MAX-RM 2000 at the scanning rate of 2° min^−1^ in a range of diffraction angle 2θ from 5° to 70°. Raman spectrum was recorded by HR800 (HORIBA spectrometer) with a wavelength of 633 nm. X-ray photoelectron spectroscopy (XPS) measurement was performed with a thermo ESCALAB 250 spectrometer. XPS data analysis was performed using Thermo Avantage software. Sorption/desorption isotherms of N_2_ at 77 K were measured by a Belsorp Max apparatus (Japan) and analyzed to evaluate specific surface areas of the composites by using the Brunauer-Emmett-Teller (BET) method, S_BET_, and pore-size distribution of the composites by density functional theory (DFT) method.

A symmetrical two-electrode supercapacitor was assembled using two pieces of the composite and a separator to investigate the electrochemical performance as an electrochemical capacitor. To achieve sufficient saturation of the electrodes and separator by the electrolyte, they were immersed in 1 M Na_2_SO_4_ solution under vacuum for 24 hrs before being assembled. Cyclic voltammetry (CV) and galvanostatic charge/discharge analysis were carried out to evaluate electrochemical performance of the electrodes with a potential window ranging from 0 to 1 V in 1 M Na_2_SO_4_ electrolyte using an electrochemical workstation (CHI 600, Shanghai Chen Hua Instrument Company). The electrochemical impedance spectroscopy (EIS) was conducted in the frequency range of 0.01 Hz ~100 kHz with perturbation amplitude of 5 mV versus the open circuit potential. The average specific capacitance was calculated according to the charge/discharge tests based on the following equation:


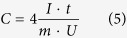


where *I*, *t*, *m* and *U* are the charge/discharge current (A), the discharge time (s), the total mass of active materials in the two electrodes (g) and the potential after the deduction of IR drop (V), respectively.

## Additional Information

**How to cite this article**: Le, T. *et al*. In-situ growth of MnO_2_ crystals under nanopore-constraint in carbon nanofibers and their electrochemical performance. *Sci. Rep.* **6**, 37368; doi: 10.1038/srep37368 (2016).

**Publisher’s note:** Springer Nature remains neutral with regard to jurisdictional claims in published maps and institutional affiliations.

## Supplementary Material

Supplementary Information

## Figures and Tables

**Table 1:** Surface areas and pore volumes for MnO_2_/CNFs composites.

Sample	S_BET_ (m^2^/g)	S_<0.7 nm_ (m^2^/g)	S_0.7~2 nm_ (m^2^/g)	S_>2 nm_ (m^2^/g)	V_total_ (cm^3^/g)	V_< 0.7 nm_ (cm^3^/g)	V_0.7~2 nm_ (cm^3^/g)	V_>2 nm_ (cm^3^/g)
MC0	603	512	79	12	0.291	0.181	0.062	0.048
MC2	645	114	526	5	0.303	0.034	0.239	0.030
MC10	649	555	89	5	0.371	0.223	0.080	0.068

S_<0.7 nm_, S_0.7~2 nm_, S_>2 nm_: surface area contributed by pores of 0~0.7 nm, of 0.7~2 nm, larger than 2 nm, respectively; V_<0.7 nm_, V_0.7~2 nm_, V_>2 nm_: pore volume contributed by pores of 0~0.7 nm, of 0.7~2 nm, larger than 2 nm, respectively.

## References

[b1] Simon P. & Gogotsi Y. Materials for electrochemical capacitors. Nat. Mater. 7, 845–854 (2008). 18956000 10.1038/nmat2297

[b2] Zhai Y. P. . Carbon materials for chemical capacitive energy storage. Adv. Mater. 23, 4828–4850 (2011). 21953940 10.1002/adma.201100984

[b3] Naoi K., Ishimoto S., Miyamoto J. I. & Naoi W. Second generation ‘nanohybrid supercapacitor’: Evolution of capacitive energy storage devices. Energy Environ. Sci. 5, 9363–9373 (2012).

[b4] Yu Z. N., Tetard L., Zhai L. & Thomas J. Supercapacitor electrode materials: Nanostructures from 0 to 3 dimensions. Energy Environ. Sci. 8, 702–730 (2015).

[b5] Zhang S. W. & Chen G. Z. Manganese oxide based materials for supercapacitors. Energy Mater. 3, 186–200 (2008).

[b6] Wei W. F., Cui X. W., Chen W. X. & Ivey D. G. Manganese oxide-based materials as electrochemical supercapacitor electrodes. Chem. Soc. Rev. 40, 1697–1721 (2011). 21173973 10.1039/c0cs00127a

[b7] Wang H. Q., Li Z. S., Huang Q. Y. & Wang X. Y. A novel hybrid supercapacitor based on spherical activated carbon and spherical MnO_2_ in a non-aqueous electrolyte. J. Mater. Chem. 10, 3883–3889 (2010).

[b8] Kim M., Hwang Y., Min K. & Kim J. Introduction of MnO_2_ nanoneedles to activated carbon to fabricate high-performance electrodes as electrochemical supercapacitors. Electrochim. Acta 113, 322–331 (2013).

[b9] Gao P. C., Lu A. H. & Li W. C. Dual functions of activated carbon in a positive electrode for MnO_2_-based hybrid supercapacitor. J. Power Sources 196, 4095–4101 (2011).

[b10] Higgins T. M. . Effect of Percolation on the Capacitance of Supercapacitor Electrodes Prepared from Composites of Manganese Dioxide Nanoplatelets and Carbon Nanotubes. ACS Nano 8, 9567–9579 (2014). 25199042 10.1021/nn5038543

[b11] Choi C. . Flexible Supercapacitor Made of Carbon Nanotube Yarn with Internal Pores. Adv. Mater. 26, 2059–2065 (2014). 24353070 10.1002/adma.201304736

[b12] Huang H. J., Zhang W. Y., Fu Y. S. & Wang X. Controlled growth of nanostructured MnO_2_ on carbon nanotubes for high-performance electrochemical capacitors. Electrochim. Acta 152, 480–488 (2015).

[b13] Wang T. . Facilitated transport channels in carbon nanotube/carbon nanofiber hierarchical composites decorated with manganese dioxide for flexible supercapacitors. J. Power Sources 274, 709–717 (2015).

[b14] Zhang D. Y., Zhang Y. H., Luo Y. S. & Chu P. K. Highly porous honeycomb manganese oxide@carbon fibers core-shell nanocables for flexible supercapacitors. Nano Energy 13, 47–57 (2015).

[b15] Zhi M. J., Manivannan A., Meng F. K. & Wu N. Q. Highly conductive electrospun carbon nanofiber/MnO_2_ coaxial nano-cables for high energy and power density supercapacitors. J. Power Sources 208, 345–353 (2012).

[b16] Wang J. G., Yang Y., Huang Z. H. & Kang F. Y. Effect of temperature on the pseudo-capacitive behavior of freestanding MnO_2_@carbon nanofibers composites electrodes in mild electrolyte. J. Power Sources 224, 86–92 (2013).

[b17] Chen L. F., Huang Z. H., Liang H. W., Guan Q. F. & Yu S. H. Bacterial-Cellulose-Derived Carbon Nanofiber@MnO_2_ and Nitrogen-Doped Carbon Nanofiber Electrode Materials: An Asymmetric Supercapacitor with High Energy and Power Density. Adv. Mater. 25, 4746–4752 (2013). 23716319 10.1002/adma.201204949

[b18] Zhu K. . *In situ* growth of MnO_2_ nanosheets on activated carbon fibers: a low-cost electrode for high performance supercapacitors. Rsc Adv. 6, 14819–14825 (2016).

[b19] Zhou D. . Freestanding MnO_2_ nanoflakes/porous carbon nanofibers for high-performance flexible supercapacitor electrodes. Electrochim. Acta 161, 427–435 (2015).

[b20] He Y. M. . Freestanding Three-Dimensional Graphene/MnO_2_ Composite Networks As Ultralight and Flexible Supercapacitor Electrodes. ACS Nano 7, 174–182 (2013). 23249211 10.1021/nn304833s

[b21] Yu G. H. . Enhancing the Supercapacitor Performance of Graphene/MnO_2_ Nanostructured Electrodes by Conductive Wrapping. Nano Lett. 11, 4438–4442 (2011). 21942427 10.1021/nl2026635

[b22] Ge J. . Facile dip coating processed graphene/MnO_2_ nanostructured sponges as high performance supercapacitor electrodes. Nano Energy 2, 505–513 (2013).

[b23] Guo W. H., Liu T. J., Jiang P. & Zhang Z. J. Free-standing porous Manganese dioxide/graphene composite films for high performance supercapacitors. J. Colloid Interface Sci. 437, 304–310 (2015). 25441365 10.1016/j.jcis.2014.08.060

[b24] Sumboja A., Foo C. Y., Wang X. & Lee P. S. Large Areal Mass, Flexible and Free‐Standing Reduced Graphene Oxide/Manganese Dioxide Paper for Asymmetric Supercapacitor Device. Adv. Mater. 25, 2809–2815 (2013). 23580421 10.1002/adma.201205064

[b25] Han G. Q. . MnO_2_ nanorods intercalating graphene oxide/polyaniline ternary composites for robust high-performance supercapacitors. Scientific Reports 4, 4824 (2014). 24769835 10.1038/srep04824 PMC4001101

[b26] Wang J. G., Kang F. Y. & Wei B. Engineering of MnO_2_-based nanocomposites for high-performance supercapacitors. Prog. Mater. Sci. 74, 51–124 (2015).

[b27] Lang X. Y., Hirata A., Fujita T. & Chen M. W. Nanoporous metal/oxide hybrid electrodes for electrochemical supercapacitors. Nat. Nanotechnology 6, 232–236 (2011). 10.1038/nnano.2011.13 21336267

[b28] Wei B. . Fabrication of manganese oxide/three-dimensional reduced graphene oxide composites as the supercapacitors by a reverse microemulsion method. Carbon 85, 249–260 (2015).

[b29] Chen W. . High-Performance Nanostructured Supercapacitors on a Sponge. Nano Lett. 11, 5165–5172 (2011). 21923166 10.1021/nl2023433

[b30] Wang J. G., Yang Y., Huang Z. H. & Kang F. Y. Coaxial carbon nanofibers/MnO_2_ nanocomposites as freestanding electrodes for high-performance electrochemical capacitors. Electrochim. Acta 56, 9240–9247 (2011).

[b31] Ma S. B. . Electrochemical properties of manganese oxide coated onto carbon nanotubes for energy-storage applications. J. Power Sources 178, 483–489 (2008).

[b32] Fischer A. E., Pettigrew K. A., Rolison D. R., Stroud R. M. & Long J. W. Incorporation of homogeneous, nanoscale MnO_2_ within ultraporous carbon structures via self-limiting electroless deposition: implications for electrochemical capacitors. Nano Lett. 7, 281–286 (2007). 17297991 10.1021/nl062263i

[b33] Yang Y., Simeon F., Hatton T. A. & Rutledge G. C. Polyacrylonitrile-based electrospun carbon paper for electrode applications. J. App. Polym. Sci. 124, 3861–3870 (2012).

[b34] Yang Y. . Highly porous electrospun polyvinylidene fluoride (pvdf)-based carbon fiber. Carbon 49, 3395–3403 (2011).

[b35] Le T. H., Yang Y., Huang Z. H. & Kang F. Y. Preparation of microporous carbon nanofibers from polyimide by using polyvinyl pyrrolidone as template and their capacitive performance. J. Power Sources 278, 683–692 (2015).

[b36] Le T. H., Yang Y., Yu L., Huang Z. H. & Kang F. Y. Polyimide‐based porous hollow carbon nanofibers for supercapacitor electrode. J. App. Polym. Sci. 133, doi: 10.1002/APP.43397 (2016).

[b37] Lowell S., Shield J. E., Thomas M. A. & Thommes M. Characterization of porous solids and powders: Surface Area. Pore Size and Density, Springer Publisher, Netherlands, 43–44 (2006).

[b38] Ferrari A. C. & Robertson J. Interpretation of Raman spectra of disordered and amorphous carbon. Physical Rev. B 61, 14095–14107 (2000).

[b39] Brousse T. . Crystalline MnO_2_ as Possible Alternatives to Amorphous Compounds in Electrochemical Supercapacitors. J. Electrochem. Soc. 153, A2171–A2180 (2006).

[b40] Li S. H., Qi L., Lu L. H. & Wang H. Y. Facile preparation and performance of mesoporous manganese oxide for supercapacitors utilizing neutral aqueous electrolytes. RSC Adv. 2, 3298–3308 (2012).

[b41] Wang J. G., Yang Y., Huang Z. H. & Kang F. Y. Rational synthesis of MnO_2_/conducting polypyrrole@carbon nanofiber triaxial nano-cables for high-performance supercapacitors. J. Mater. Chem. 33, 16943–16949 (2012).

[b42] Toupin M., Brousse T. & Bélanger D. Charge Storage Mechanism of MnO_2_ Electrode Used in Aqueous Electrochemical Capacitor. Chem. Mater. 16, 3184–3190 (2004).

[b43] Lei Z. B., Zhang J. T. & Zhao X. S. Ultrathin MnO_2_ Nanofibers Grown on Graphitic Carbon Spheres as High-performance Asymmetric Supercapacitor Electrodes. J. Mater. Chem. 22, 153–160 (2012).

[b44] He S. J., Hu C. X., Hou H. Q. & Chen W. Ultrathin MnO_2_ nanosheets supported on cellulose based carbon papers for high-power supercapacitors. J. Power Sources 246, 754–761 (2014).

[b45] Zhang Y. F., Zhang C. X., Huang G. X., Xing B. L. & Duan Y. L. Synthesis and Capacitive Properties of Manganese Oxide Nanoparticles Dispersed on Hierarchical Porous Carbons. Electrochim. Acta 166, 107–116 (2015).

[b46] Wu Z. S. . High-energy MnO_2_ nanowire/graphene and graphene asymmetric electrochemical capacitors. Acs Nano 10, 5835–5842 (2010). 10.1021/nn101754k 20857919

[b47] Cheng Y. W., Lu S. T., Zhang H. B., Varanasi C. V. & Liu J. Synergistic Effects from Graphene and Carbon Nanotubes Enable Flexible and Robust Electrodes for High-Performance Supercapacitors. Nano Lett. 12, 4206–4211 (2012). 22823066 10.1021/nl301804c

[b48] Li P. X. . Core-Double-Shell, CNT@Polypyrrole@MnO_2_ Sponge as Freestanding, Compressible Supercapacitor Electrode. Appl. Mater. Interfaces 6, 5228–5234 (2014). 10.1021/am500579c 24621200

[b49] Zhao L. . Honeycomb porous MnO_2_ nanofibers assembled from radially grown nanosheets for aqueous supercapacitors with high working voltage and energy density. Nano Energy 4, 39–48 (2014).

[b50] Chou T. C., Doong R. A., Hu C. C., Zhang B. S. & Su D. S. Hierarchically Porous Carbon with Manganese Oxides as Highly Efficient Electrode for Asymmetric Supercapacitors. Chemsuschem 7, 841–847 (2014). 24504702 10.1002/cssc.201301014

[b51] Lee D. G., Kim B. & MnO_2_ decorated on electrospun carbon nanofiber/graphene composites as supercapacitor electrode materials. Synthetic Met. 219, 115–123 (2016).

[b52] Lee D. G., Ji H. K. & Kim B. H. Hierarchical porous MnO_2_/carbon nanofiber composites with hollow cores for high-performance supercapacitor electrodes: Effect of poly (methyl methacrylate) concentration. Electrochim. Acta 200, 174–181 (2016).

[b53] Ko W. Y., Chen Y. F., Lu K. M. & Lin K. J. Porous honeycomb structures formed from interconnected MnO_2_ sheets on CNT-coated substrates for flexible all-solid-state supercapacitors. Scientific Reports 6, 18887 (2016). 26726724 10.1038/srep18887 PMC4750103

[b54] Hung C. J., Lin P. & Tseng T. Y. High energy density asymmetric pseudocapacitors fabricated by graphene/carbon nanotube/MnO_2_, plus carbon nanotubes nanocomposites electrode. J. Power Sources 259, 145–153 (2014).

[b55] Ning P. . Facile synthesis of carbon nanofibers/MnO_2_, nanosheets as high-performance electrodes for asymmetric supercapacitors. Electrochim. Acta 210, 754–761 (2016).

[b56] Fic K., Lota G., Meller M. & Frackowiak E. Novel insight into neutral medium as electrolyte for high-voltage supercapacitors. Energy Environ. Sci. 5, 5842–5850 (2012).

[b57] Hsu Y. K., Chen Y. C., Lin Y. G. & Chen K. H. High-cell-voltage supercapacitor of carbon nanotube/carbon cloth operating in neutral aqueous solution. J. Mater. Chem. 22, 3383–3387 (2012).

