# Supplementary material for: In-situ growth of MnO2 crystals under nanopore-constraint in carbon nanofibers and their electrochemical performance
Source: Sci Rep. 2016 Nov 21;6:37368. doi: 10.1038/srep37368 (PMC5116767; doi:10.1038/srep37368)
Supplement: Supplementary Information [file srep37368-s1.doc]

**Supplementary Information:**

**In-situ growth of MnO_2_ crystals under nanopore-constraint in carbon nanofibers and their electrochemical performance**

TrungHieu Le^a^, Ying Yang^a *^, Liu Yu^a^, Zhenghong Huang^b^, and Feiyu Kang^b^

^a^ State Key Laboratory of Control and Simulation of Power System and Generation Equipments，Tsinghua University, Beijing 100084, China

^b^ Laboratory of Advanced Materials, Department of Materials Science and Engineering, Tsinghua University, Beijing 100084, China

**Figure S1 Cyclic Voltammetry (CV) curves of the MC composites at the scan rate of (a) 20 mV s^-1^, (b) 50 mV s^-1^ and (c) 100 mV s^-1^, (d) charge/discharge curves of the MC composites at the current density of 10 A g^-1^.**


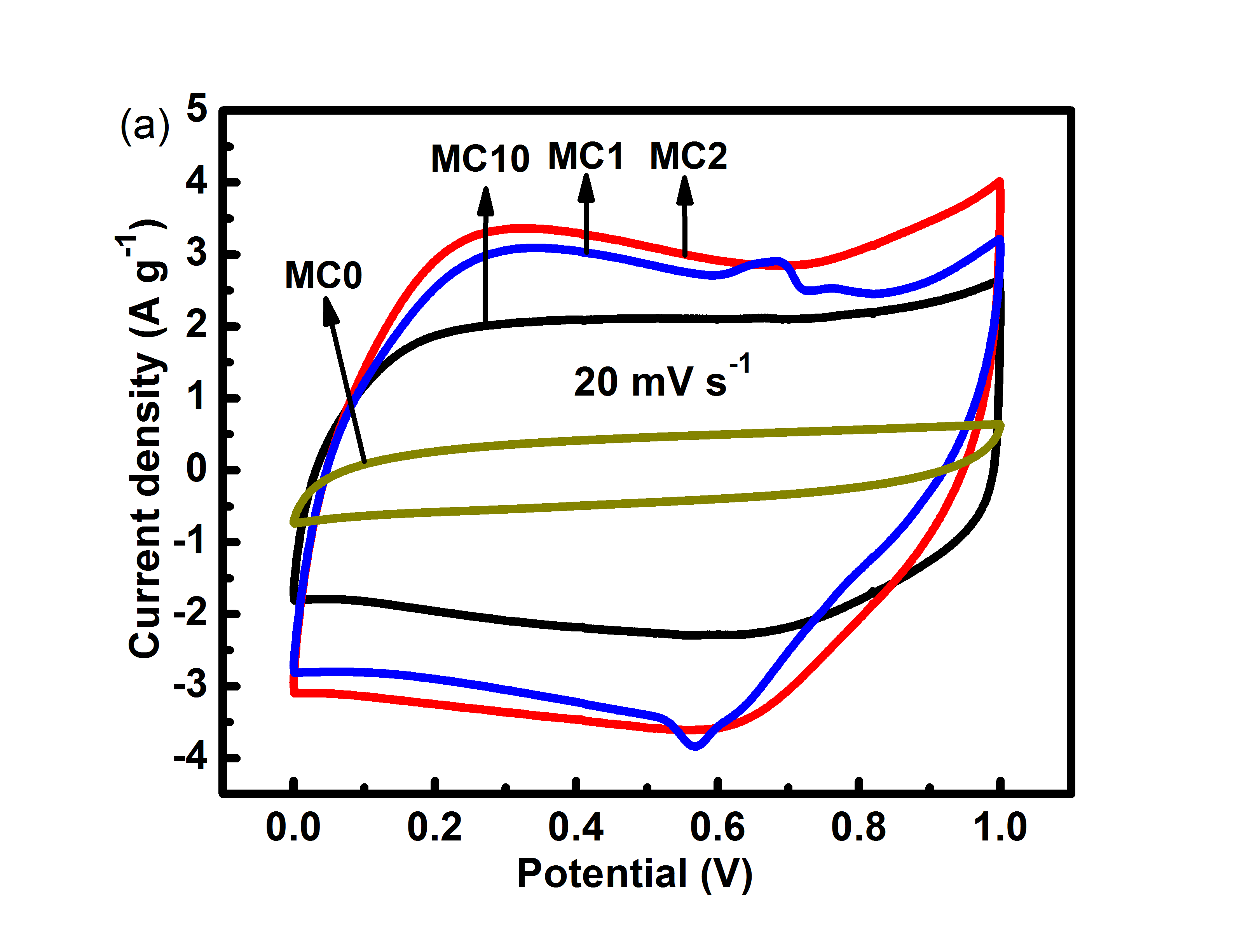

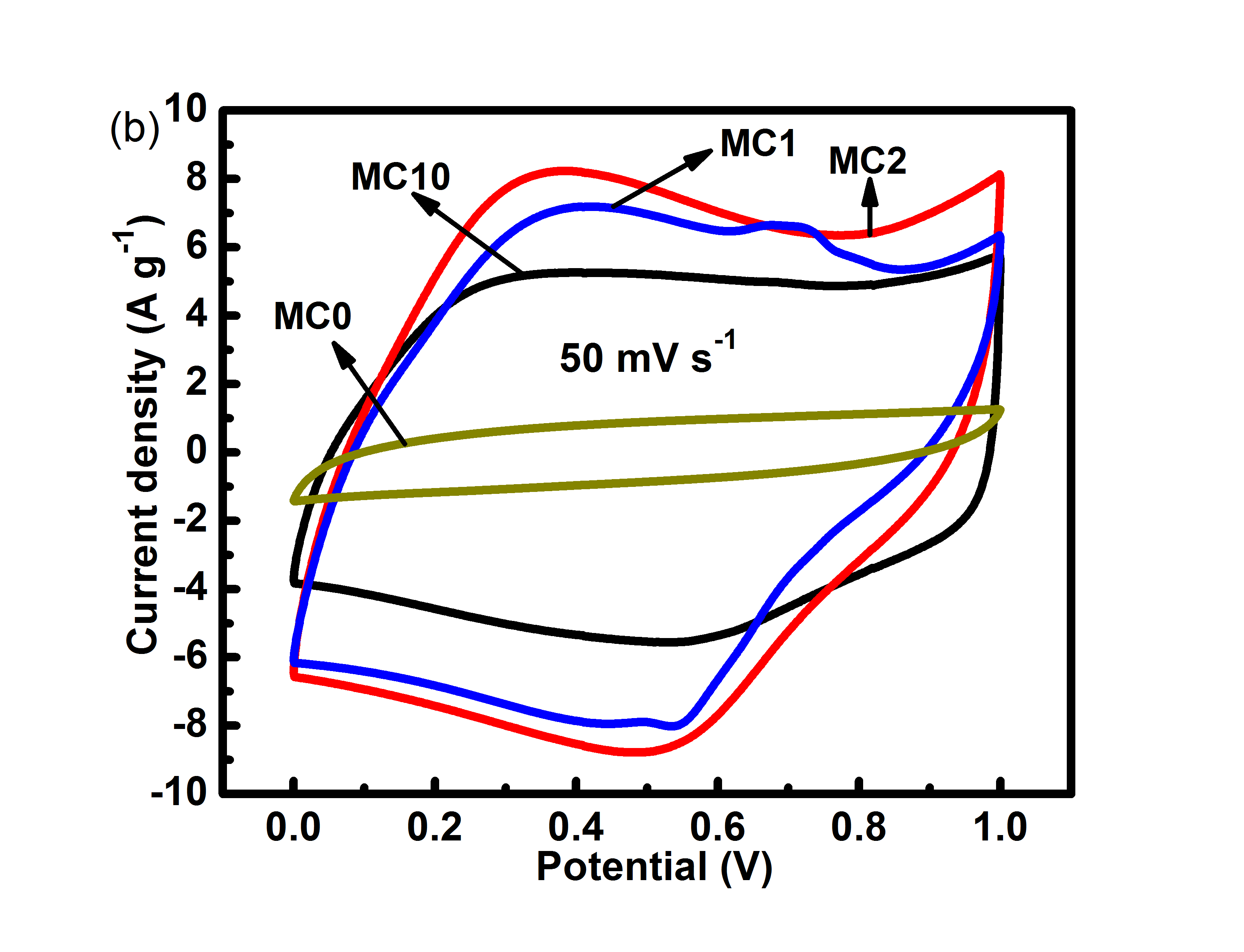


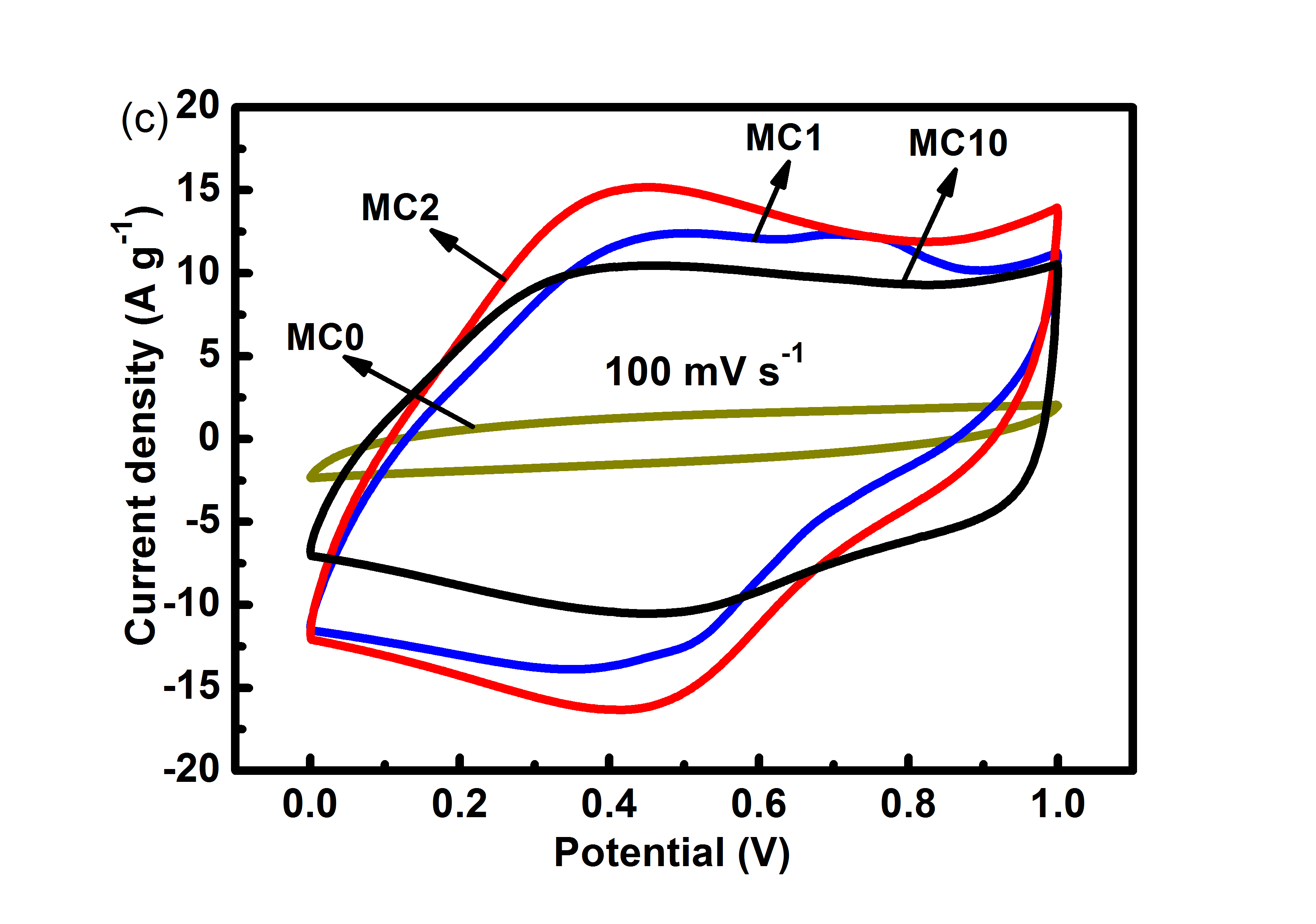

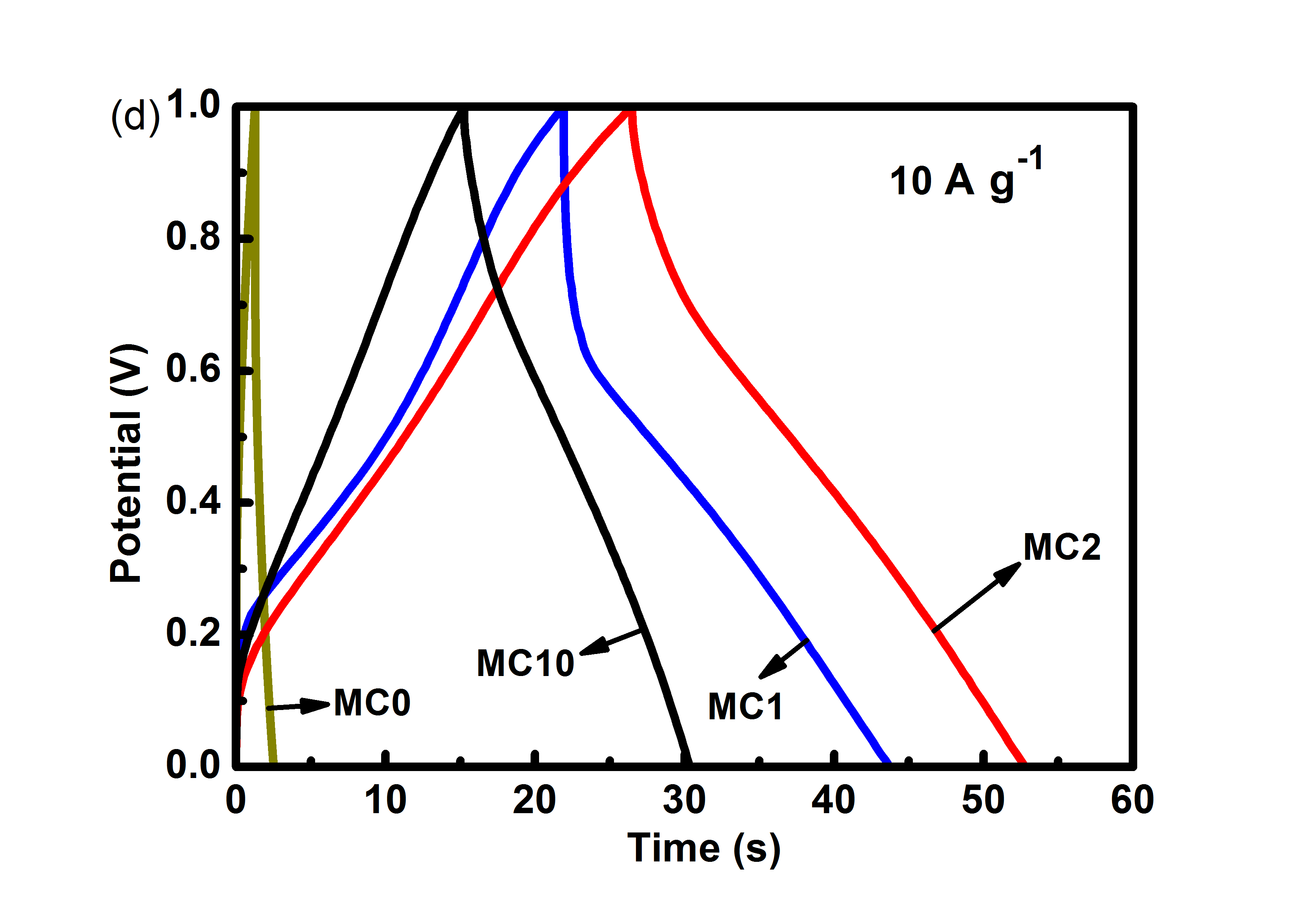


**Table S1: Comparison of High Capacitance MnO_2_ Composite Electrodes via different methodologies**

| Substrate | Technique | Mass loading | S_BET_  m^2^ g^-1^ | SC  F g^-1^ | Rate capability | Binder free and flexible | Cost/Process | Ref. |
| --- | --- | --- | --- | --- | --- | --- | --- | --- |
| CNTs-Sponge | ED | 12% | 174 | 1230/1 mV s^-1^ | 200/100 mV s^-1^ | no | Moderate/Moderate | [29] |
| Nanoporous Au | ED | 41% | / | 1145/50 mV s^-1^ | 825/100 mV s^-1^ | yes | High/Moderate | [27] |
| Au-PET | ED | 20% | / | 1130/2 mV s^-1^ | 600/100 mV s^-1^ | no | High/Complex | [S1] |
| Ni dendrites | ED | 0.35 mg cm^-2^ | 23.4 | 1125/5 mV s^-1^ | 566/200 mV s^-1^ | no | High/Complex | [S2] |
| Carbon paper | ED | 5.4% | 98 | 1149/1 A g^-1^ | 565/100 A g^-1^ | yes | Low/Moderate | [S3] |
| G-CNTs | EPD | / | / | 964/1 A g^-1^ | 644/10 A g^-1^ | no | Moderate/Complex | [55] |
| Carbon-Ni | Coat | 6 mg cm^-2^ | / | 1301 | / | no | Moderate/Simple | [S4] |
| MnO2/G/PCNFs | TO | / | 699 | 210 /1 mA cm^-2^ | 81%/20 mA cm^-2^ | yes | Low/Simple | [52] |
| MnO2/HCNFs | TO | 6.11% | / | 228 /1 mA cm^-2^ | 83%/20 mA cm^-2^ | yes | Low/Simple | [53] |
| HOPC | RD | 10.8% | 437 | 1312/25 mV s^-1^ | 75%/100 mV s^-1^ | no | Low/Simple | [50] |
| Carbon foam | RD | 3.4% | 69.6 | 1270/0.5 A g^-1^ | 500/20 A g^-1^ | yes | Moderate/Moderate | [S5] |

G: Graphene; CNTs: Carbon nanotubes; CNFs: Carbon nanofibers; PCNFs: Porous carbon nanofibers; HCNFs: Hollow carbon nanofibers;

HOPC: Hierarchically ordered Porous Carbons; PET: Polyethylene terephthalate; AAI: Iron acetylacetonate

ED: Electrochemical deposition; EPD: Electrophoretic Deposition; RD: Redox deposition; TO: Thermal Oxidation

**Table S2: Comparison of MnO_2_/CNF Composite Electrodes**

| Electrode | Mass loading | SC (F g^-1^) | Rate capability | Ref. |
| --- | --- | --- | --- | --- |
| MnO_2_/CNTs-CNFs | 0.33 mg | 517/5 mV s^-1^ | 111/200 mV s^-1^ | [13] |
| MnO_2_/CNFs | / | 295/0.1 A g^-1^ | 58/0.5 A g^-1^ | [14] |
| MnO_2_/AAI-CNFs | 23% | 900/2 mV s^-1^ | / | [15] |
| MnO_2_/PCNFs | 5.7% | 520/0.5 A g^-1^ | 230/20 A g^-1^ | [19] |
| MnO_2_/CNFs | 0.33 mg cm^-2^ | 557/1 A g^-1^ | 213/100 mV s^-1^ | [30] |
| MnO_2_/CNFs | / | 292/2.5 A g^-1^ | 170/8 A g^-1^ | [49] |
| MnO_2_/G-PCNFs | / | 210/1 mA cm^-2^ | 81%/20 mA cm^-2^ | [52] |
| MnO_2_/HCNFs | 6.11% | 228/1 mA cm^-2^ | 83%/20 mA cm^-2^ | [53] |
| MnO_2_/CNFs | 74.5% | 151/1 A g^-1^ | 115/8 A g^-1^ | [56] |
| MnO_2_/CNTs-CNFs | 1.5 mg cm^-2^ | 374/2 mV s^-1^ | 198/100 mV s^-1^ | [S6] |
| MnO_2_/HCNFs | / | 855/2 A g^-1^ | 81%/100 mV s^-1^ | [S7] |
| MnO_2_/HCNFs | / | 237/10 mV s^-1^ | 100/100 mV s^-1^ | [S8] |
| MnO_2_/PCNFs | 11% | 1282/0.2 A g^-1^ | 400/50 A g^-1^ | This work |

G: Graphene; CNTs: Carbon nanotubes; CNFs: Carbon nanofibers; PCNFs: Porous carbon nanofibers; HCNFs: Hollow carbon nanofibers; AAI: Iron acetylacetonate

**Table S3: Comparison of MnO_2_/Carbon Composite Electrodes shown in Ragon plot**

| Substrate | Technique | Mass loading | Structure and Mophology  Of MnO_2_ | MnO_2_ Phase | S_BET_  (m^2^ g^-1^) | Binder free | Electrolyte  (Potential window) | SC_max_  (F g^-1^) | Rate capability | Ref. |
| --- | --- | --- | --- | --- | --- | --- | --- | --- | --- | --- |
| CNTs | RD | / | Nanoparticles (20~30nm) | / | 159.8 | no | 1M Na_2_SO_4_  (0-1V) | 248/0.15 A g^-1^ | 214/ 2 A g^-1^ | [12] |
| CNTs-CNFs | RD | 0.33 mg | 20-nm-thick flaky MnO_2_ nanostructures | α-MnO_2_ | 18.67 | yes | 1M Na_2_SO_4_  (0-1V) | 517/5 mV s^-1^ | 112/200 mV s^-1^ | [13] |
| CFC | RD | / | Porous honeycomb-like MnO_2_ (~400nm) | δ-MnO_2_ | / | yes | 1M Na_2_SO_4_  (0-1.6V) | 295/0.1 A g^-1^ | 58/0.5 A g^-1^ | [14] |
| G | ED | 11.7% | Nanosheet (40~50nm) | amorphous | 281 | yes | 0.5M Na_2_SO_4_  (0-1V) | 465/2 mV s^-1^ | / | [20] |
| CP | RD | 7.9% | Nanosheet (a few nanometers in thickness) | δ-MnO_2_ | 61.9 | yes | 1M Na_2_SO_4_  (0-0.7V) | 307/0.5 A g^-1^ | 155/300 A g^-1^ | [44] |
| HPCs | RD | 22% | Nanoparticles (200~300nm) | δ-MnO_2_ | 532 | no | 3M KOH  (0-0.9V) | 167/0.05 A g^-1^ | 124/5 A g^-1^ | [45] |
| G | RD | 21.7% | Nanowires (diameters 10-20nm) | α-MnO_2_ | 107 | no | 1M Na_2_SO_4_  (0-1V) | / | / | [46] |
| G-CNTs | RD | 0.4 mg cm^-2^ | Nanosheet (100~200nm) | amorphous | / | yes | 1M Na_2_SO_4_  (0-1V) | 326/10 mV s^-1^ | 148/500 mV s^-1^ | [47] |
| CNFs | RD | / | Nanosheets (~20nm) (thickness 3~7nm) | δ-MnO_2_ | / | yes | 1M Na_2_SO_4_  (0-2.2V) | 292/2.5 A g^-1^ | 170/8 A g^-1^ | [49] |
| HPCs | RD | 10.8% | Porous structures; crystals 3~5nm | δ-MnO_2_ | 437 | no | 1M Na_2_SO_4_  (0-0.9V) | 1312/25 mV s^-1^ | 75%/100 mV s^-1^ | [50] |
| G-CNFs | TO | / | Nanoparticles (50~100nm) | / | 699 | yes | 6M KOH  (0-1V) | 210/1 mA cm^-2^ | 81%/20 mA cm^-2^ | [52] |
| CNFs | TO | 6.11% | Nanosheets (~50nm) | / | / | yes | 6M KOH  (0-1V) | 228/1 mA cm^-2^ | 83%/20 mA cm^-2^ | [53] |
| PCNFs | RD | 11% | Ultrathin MnO_2_ (thickness 2~5 nm) | α-MnO_2_ | 645 | yes | 1M Na_2_SO_4_  (0-1V) | 1282/0.2 A g^-1^ | 400/50 A g^-1^ | This work |

CP: Carbon papers; CFC: Carbon fiber cloth; G: Graphene; CNTs: Carbon nanotubes; CNFs: Carbon nanofibers; PCNFs: Porous carbon nanofibers; HPCs: Hierarchically Porous Carbons.

ED: Electrochemical deposition; RD: Redox deposition; TO: Thermal Oxidation

**References**

[S1] Chen, Y. L., Chen, P. C., Chen, T. L., Lee, C. Y., Chiu, H. T. Nanosized MnO_2_ spines on Au stems for high-performance flexible supercapacitor electrodes. J. Mater. Chem. A **42**, 13301-13307 (2013).

[S2] Sun, Z., Firdoz, S., Yap, E. Y., Lu, X. Hierarchically structured MnO_2_ nanowires supported on hollow Ni dendrites for high-performance supercapacitors. Nanoscale **10**, 4379-87 (2013).

[S3] Anothumakkool, B., Kurungot, S. Electrochemically grown nanoporous MnO_2_ nanowalls on a porous carbon substrate with enhanced capacitance through faster ionic and electrical mobility. Chem. Commun. **50**, 7188-90 (2014).

[S4] Zhi, J., Reiser, O., Wang, Y., Hu, A. A high performance flexible all solid state supercapacitor based on the MnO_2_ sphere coated macro/mesoporous Ni/C electrode and ionic conducting electrolyte. Nanoscale **8**, 11976-11983 (2016).

[S5] He, S. J., Chen, W. High performance supercapacitors based on three-dimensional ultralight flexible manganese oxide nanosheets/carbon foam composites. J. Power Sources **262**, 391-400 (2014).

[S6] Wang, J. G., Yang, Y., Huang, Z. H., Kang, F. Y. Synthesis and electrochemical performance of MnO_2_/CNTs-embedded carbon nanofibers nanocomposites for supercapacitors. Electrochim. Acta **75**, 213-219 (2012).

[S7] Jun, J., Lee, J. S., Shin, D. H., Kim, S. G., Jang, J. Multidimensional MnO_2_ nanohair-decorated hybrid multichannel carbon nanofiber as an electrode material for high-performance supercapacitors. Nanoscale **7**, 16026-16033 (2015).

[S8] Hong, S., Lee, S., Paik, U. Core-Shell Tubular Nanostructured Electrode of Hollow Carbon Nanofiber/Manganese Oxide for Electrochemical Capacitors. Electrochim. Acta **141**, 39-44 (2014).
